# Identification of Four Biomarkers of Human Skin Aging by Comprehensive Single Cell Transcriptome, Transcriptome, and Proteomics

**DOI:** 10.3389/fgene.2022.881051

**Published:** 2022-08-23

**Authors:** Rui Mao, Yunying Wang, Fan Wang, Lei Zhou, Sha Yan, Shanshan Lu, Wei Shi, Yiya Zhang

**Affiliations:** ^1^ Department of Dermatology, Xiangya Hospital, Central South University, Changsha, China; ^2^ Hunan Key Laboratory of Aging Biology, Xiangya Hospital, Central South University, Changsha, China; ^3^ Department of Dermatology, The Second Affiliated Hospital of Xinjiang Medical University, Urumqi, China; ^4^ Research Center of Carcinogenesis and Targeted Therapy, Xiangya Hospital, Central South University, Changsha, China; ^5^ The Higher Educational Key Laboratory for Cancer Proteomics and Translational Medicine of Hunan Province, Xiangya Hospital, Central South University, Changsha, China; ^6^ National Clinical Research Center for Geriatric Disorders, Xiangya Hospital, Central South University, Changsha, China

**Keywords:** single cell transcriptome analysis, transcriptome, proteomics, skin aging, senescence, fibroblast

## Abstract

**Background:** Aging is characterized by the gradual loss of physiological integrity, resulting in impaired function and easier death. This deterioration is a major risk factor for major human pathological diseases, including cancer, diabetes, cardiovascular disease and neurodegenerative diseases. It is very important to find biomarkers that can prevent aging.

**Methods:** Q-Exactive-MS was used for proteomic detection of young and senescence fibroblast. The key senescence-related molecules (SRMs) were identified by integrating transcriptome and proteomics from aging tissue/cells, and the correlation between these differentially expressed genes and well-known aging-related pathways. Next, we validated the expression of these molecules using qPCR, and explored the correlation between them and immune infiltrating cells. Finally, the enriched pathways of the genes significantly related to the four differential genes were identified using the single cell transcriptome.

**Results:** we first combined proteomics and transcriptome to identified four *SRMs*. Data sets including GSE63577, GSE64553, GSE18876, GSE85358, and qPCR confirmed that ETF1, PLBD2, ASAH1, and MOXD1 were identified as SRMs. Then the correlation between SRMs and aging-related pathways was excavated and verified. Next, we verified the expression of SRMs at the tissue level and qPCR, and explored the correlation between them and immune infiltrating cells. Finally, at the single-cell transcriptome level, we verified their expression and explored the possible pathway by which they lead to aging. Briefly, ETF1 may affect the changes of inflammatory factors such as IL-17, IL-6, and NFKB1 by indirectly regulating the enrichment and differentiation of immune cells. MOXD1 may regulate senescence by affecting the WNT pathway and changing the cell cycle. ASAH1 may affect development and regulate the phenotype of aging by affecting cell cycle-related genes.

**Conclusion:** In general, based on the analysis of proteomics and transcriptome, we identified four SRMs that may affect aging and speculated their possible mechanisms, which provides a new target for preventing aging, especially skin aging.

## Introduction

The physiological changes in human aging are mainly reflected in the loss of tissue cells and constituent substances, the slowdown of metabolic rate, and the decrease of the function of body and organs. *In vivo*, senescence is an important mechanism to prevent damaged cells from transforming into tumor cells (Baker et al., 2016), and plays an important physiological role in wound healing (Demaria et al., 2014). The long-term existence of senescent cells and their secretory components in tissues can lead to aging-related tissue decline, and can even be used as a factor in promoting tumorigenesis (Tchkonia et al., 2013). Skin aging is the most obvious manifestation of body aging, which can be used as a predictor of life expectancy and health. The main physiological changes of skin aging are decreased skin elasticity, wrinkles and age spots. The human desire for lasting beauty has further aroused people’s interest in this topic, so a lot of means and efforts have been invested in basic and applied research to study the mechanism of skin aging (Gruber et al., 2020).

Fibroblasts are the main cellular components in the dermis. Collagen fibers, elastic fibers, and matrix components secreted by fibroblasts constitute the main body of the dermis together with fibroblasts. A large number of studies have proved that fibroblasts play an important role in the process of skin aging because of their unique biological characteristics. Replicative aging of fibroblasts *in vitro* has often been used as a cell model for aging (Maier and Westendorp, 2009).

Genomic instability, telomere wear, epigenetic changes, loss of protein balance, nutritional perception disorders, mitochondrial dysfunction, cell aging, stem cell depletion, and changes in intercellular communication are major characteristics representing the common ground of aging in different organisms (López-Otín et al., 2013). A major challenge is to dissect the relationship between candidate features and their relative contribution to aging. Disorders of the immune system can lead to significant changes in aging-related intercellular communication, known as inflammaging (Salminen et al., 2012), which is accompanied by the accumulation of pro-inflammatory phenotypes in mammals. On the other hand, the function of adaptive immune system is decreased, which occurs in parallel with inflammatory senescence (Deeks, 2011). Immunosenescence can aggravate the aging phenotype at the systemic level, which is due to the inability of immune system disorders to eliminate infectious factors, infected cells and precancerous cells. Another function of immune cells is to recognize and remove senescent cells and hyperploid cells, which accumulate in aging tissues and precancerous lesions (Senovilla et al., 2012; Cabreiro et al., 2013). However, the relationship between immunity and aging and the mechanism of interaction still needs to be further explored.

**FIGURE 1:**
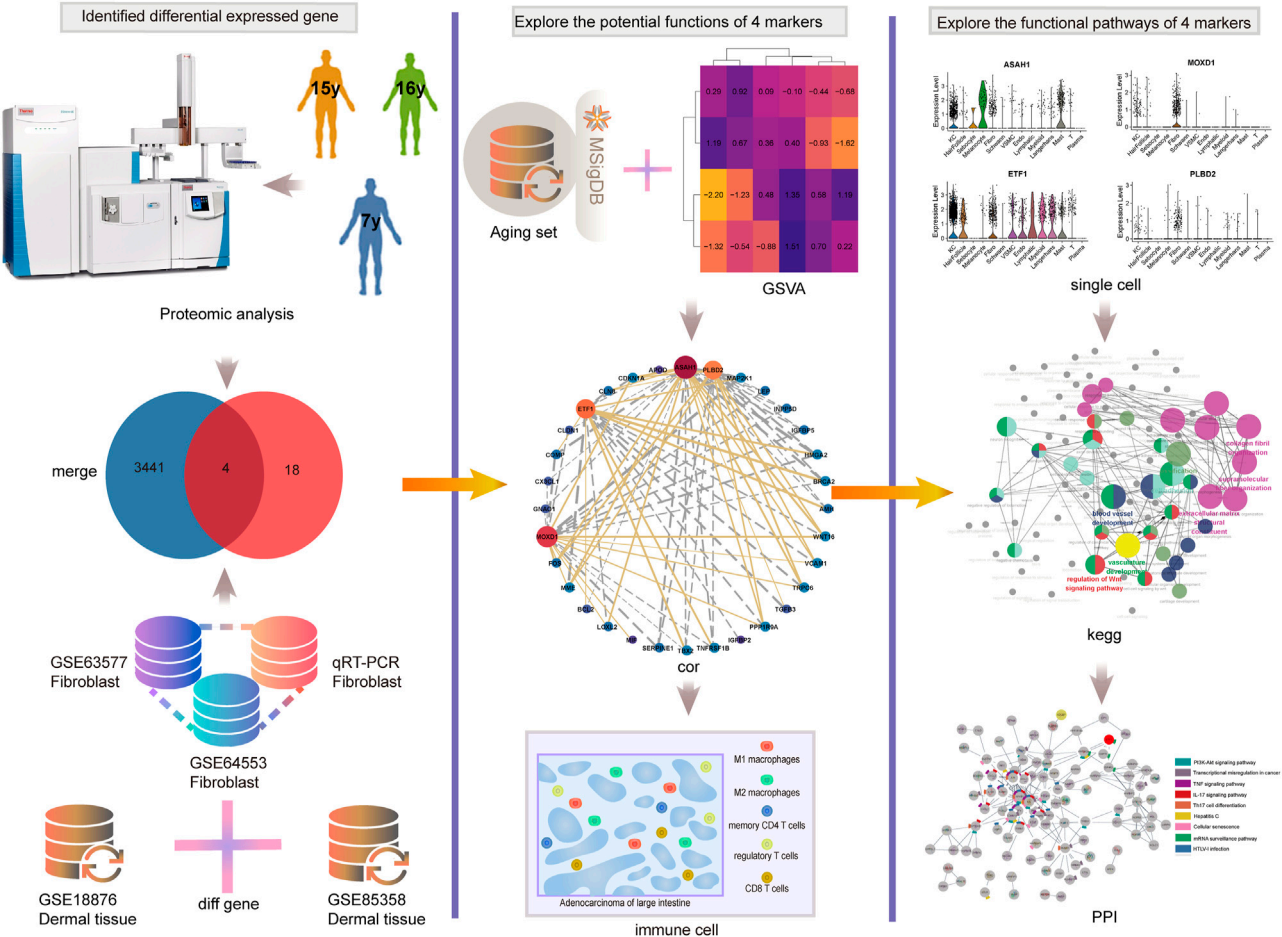
The analysis process of this study. We first combined proteomics and transcriptome to identified four key *SRMs*. Then the correlation between *SRMs* and aging-related pathways was excavated and validated. Next, we validated the expression of *SRMs* at the tissue level and qPCR, and explored the correlation between them and immune infiltrating cells. Finally, at the single-cell transcriptome level, we validated their expression and explored the possible pathway by which they lead to aging.

Therefore, exploring the new mechanism of aging and exploring the biomarkers of aging is very important for delaying senescence. In addition, the study of the relationship between aging and immunity can further clarify the mechanism of aging. The analysis process of this study is shown in Figure 1. Here, combined with the comprehensive analysis of proteomics, single cell transcriptome, and transcriptome, we identified four new aging markers and the correlation between *SRMs* and aging-related pathways would be excavated and verified. we would validate the expression of *SRMs* at the tissue level and qPCR, and explored the correlation between them and immune infiltrating cells to elucidated their possible mechanisms leading to aging.

## Methods and Materia

### Cell Culture

Primary normal human foreskin fibroblasts were isolated 3 donors (7 years donor, 15 years donor and 16 years donor) and cultured in DMEM (Gibco; Waltham, MA, United States) with 10% FBS (Gibco; Waltham, MA, United States) as previously described (Xie et al., 2021). The young (6∼8 **passage**) and **senescent** (34∼42 **passage**) fibroblasts were verified using SA-b-Gal staining and then used for further analysis. Informed consent to participate in the study have been obtained from participants (or their parent or legal guardian in the case of children under 16 or illiterate participants). Three human foreskin specimens involved in this study were collected in June 2021.

### qPCR Analysis

The RNA of young and **senescent** fibroblasts was collected using TRIzol (Invitrogen). 2 μg RNA was used for reverse transcription (Maxima H Minus First Strand cDNA Synthesis Kit with dsDNase, ThermoScience, K1682, United States), and gene expression was analyzed using an Applied Biosystems ® 7,500 machine (Life Technologies, United States). The primers were shown in Table 1.

**TABLE 1:** Primer Sequences for qRT-PCR.

Gene Name	Forward Primer	Reverse Primer
ASAH1	5'- AGA​TGT​CAT​GTG​GAT​AGG​GTT​CC -3'	3'- GGG​GCC​AAT​ATC​TTG​GTC​TTG -5'
ETF1	5'- CAC​GAG​TGG​CAA​AAA​TGT​TAG​C -3'	3'- CCA​GGA​CTG​AAA​GGC​GGT​TTA -5'
MOXD1	5'- AGA​GTT​TGC​GGT​TAT​TGA​ATC​CT -3'	3'- CGC​TGT​CGT​TAA​AGT​TGT​TGC -5'
PLBD2	5'- GGC​CCC​TTC​GAG​TAT​GAA​GTC -3'	3'- TCC​TGC​ATC​CAC​TCT​AGG​TTG -5'
GAPDH	5'- GAA​AGC​CTG​CCG​GTG​ACT​AA -3'	3'- GCC​CAA​TAC​GAC​CAA​ATC​AGA​G -5'

qRT-PCR, quantitative real-time polymerase chain reaction.

### Proteomic Analysis

The whole-cell extracts of young and senescent fibroblasts were prepared in NP40 buffer (Beyotime). After separation on SDS-PAGE, the protein from different bands were excised and used for trypsin digestion. The peptides were separated using an Ultimate 3,000 RSLCnano system and then analyzed on the Q Exactive (Thermo Fischer Scientific, San Jose, CA, United States). Proteome discoverer 1.4 (PD1.4, Thermo Fisher Scientific) with Masccot was used for protein identification, as previously described (Huang et al., 2010). The tandem mass spectrometry data was converted into a PKL file using Masslynx v 4.0 software (Micromass, Waters, United States), and then was imported into an local Mascot 2.1 search engine (Matrix Science Ltd., London, UK), which was licensed to Hunan Normal University, Changsha, China (KA108-2433), against the National Center for Biotechnology nonredundant database (NCBInr 2008.09.07; 7020262 sequences). The search parameters were as follows: the enzyme was trypsin; the taxonomy was selected as Homo sapiens; the mass tolerance was ±0.3 Da; the MS/MS tolerance was ±0.3 Da; the peptide charge is 1+, 2+ and 3+; the missed cleavage sites were allowed up to 1; the fixed modifications were selected as carboxymethyl (cysteine); the variable modification was selected as oxidation (methylation) or none; the data format was selected as micromass PKL format; and the instrument was selected as ESI-Q-TOF. Individual ions scores >31 indicate identity or extensive homology (*p* < 0.05), and were considered significant. But for those protein matching unique peptides, individual ions scores >38 indicate identity or extensive homology (*p* < 0.01) were considered significant (Huang et al., 2010). The results after data standardization obtained from proteomic sequencing are stored in a supplementary file named “ Supplementary Table S1”.

### Data Acquisition and Processing

Gene Expression Omnibus (GEO) database stores curated gene expression DataSets, as well as original Series and Platform records in the GEO repository. Data sets such as GSE63577, GSE64553, GSE151177, GSE85358, and GSE18876 are downloaded on the GEO database (https://www.ncbi.nlm.nih.gov/geo/). The GSE85358 and GSE18876 matrices are merged into large matrices using R language, and the batch effect is removed by using the “combat” function of the R software “sva” package (Leek et al., 2012) (version 3.45.0). The aging gene set was downloaded from the MSigDB database (https://www.gsea-msigdb.org/gsea/msigdb/index.jsp).

### Principal Component Analysis

Principal component analysis (Principal Component Analysis, PCA) is a reduced-dimensional data processing method. We use the “prcomp” function in the “stats” package (version 3.6.1) of R language for PCA analysis, and then use the “scale” function to normalize z-score. Finally, the visualization is carried out by using the “scatterplot3d” package (version 0.3.41) in R software.

### Venn and Upset Analysis

We conducted Venn analysis by using the “venn.diagram” function of the “VennDiagram” package (Chen and Boutros, 2011) (version 1.6.20), and then use the “calculate.overlap” function to calculate the duplicates between each data set. The “Upset” analysis is done by executing the “Upset” function in the “UpSetR” package (Conway et al., 2017) (version 1.4.0), and the venn circle is drawn by using the “ggvenn” function in the “yyplot” package (version 0.0.8).

### Gene Difference Analysis

In this study, we used the “limma” (Robinson et al., 2010) package (version 3.50.3) of R software to screen differentially expressed genes. The screening criteria was that the adjusted-P value was less than 0.05 and the absolute value of Fold Change (FC) was greater than 2. Advanced volcano plot was performed using the OmicStudio tools at https://www.omicstudio.cn/tool.

### GSVA and Enrichment Analysis

Gene set variation analysis is a non-parametric and unsupervised algorithm. Input the chip data standardized by log2 as gene expression matrix, and then input a specific gene set. The GSVA enrichment score is calculated by “GSVA” (Hänzelmann et al., 2013) package (version 1.42.0) and “GSEABase” package (version 1.56.0). The final output is the data matrix corresponding to each sample with each gene set. Drawing heat map of correlation coefficient using “pheatmap” package. The enrichment analysis of GO and KEGG is carried out by using the “ClueGO (Bindea et al., 2009)” plug-in in Cytoscape (V3.8.2), and the visual adjustment is made by “CluePedia”. We exclude the terms that the *p* value in the enrichment pathway is less than 0.05 or that the enrichment pathway contains less than 3 genes.

### Single Cell Data Analysis

We annotated and analyzed the GSE151177 dataset using the Seurat package (version 4.0.6) (Hao et al., 2021). In the quality control stage, we filtered out the cells with the minimum number of expressed genes less than 300 and the genes with the minimum number of expressed cells less than 4. Then we conducted the second screening according to the conditions that the proportion of mitochondrial genes is less than 50% and the proportion of ribosomal genes is more than 3%. Finally, we filtered out the housekeeping gene and scored the cell cycle.

### Protein-Protein Interaction and Correlation Analysis

We did a PPI analysis using the STRING (V11.5) online website, setting minimum required interaction score as 0.4. Enrichment analysis and network visualization are carried out by using “string” and “string enrichment” plug-ins in Cytoscape (V3.8.2) (Shannon et al., 2003) software. In addition, we use stats package to calculate the person correlation coefficient between the two expression matrices. And the “igraph” package (version 1.2.6) is used to visualize the correlation network.

### Estimation of Immune Cell Abundance

Based on the matrix of GSE18876 and GSE85358, we calculated the abundance of immune cells by using Cibersort (https://cibersortx.stanford.edu/index.php) (Newman et al., 2015), MCP Counter (version 1.1) (Becht et al., 2016), and Xcell (https://xcell.ucsf.edu/) (Aran et al., 2017) software packages, and excluded the samples with *p* < 0.05 among them.

### Ethics Approval and Consent to Participate

All experiments were performed in accordance with relevant guidelines and regulations. All the experiments in this study were approved by the Clinical Medical Ethics Committee of Xiangya Hospital of Central South University (202103574).

### Data Statistics

All the analyses in this study are carried out using R software (version 4.1.2), and all the codes used are stored in the Supplementary Table S2.

## Results

### 18 Differentially Expressed Proteins Were Identified by Qualitative Proteomics

**FIGURE 2:**
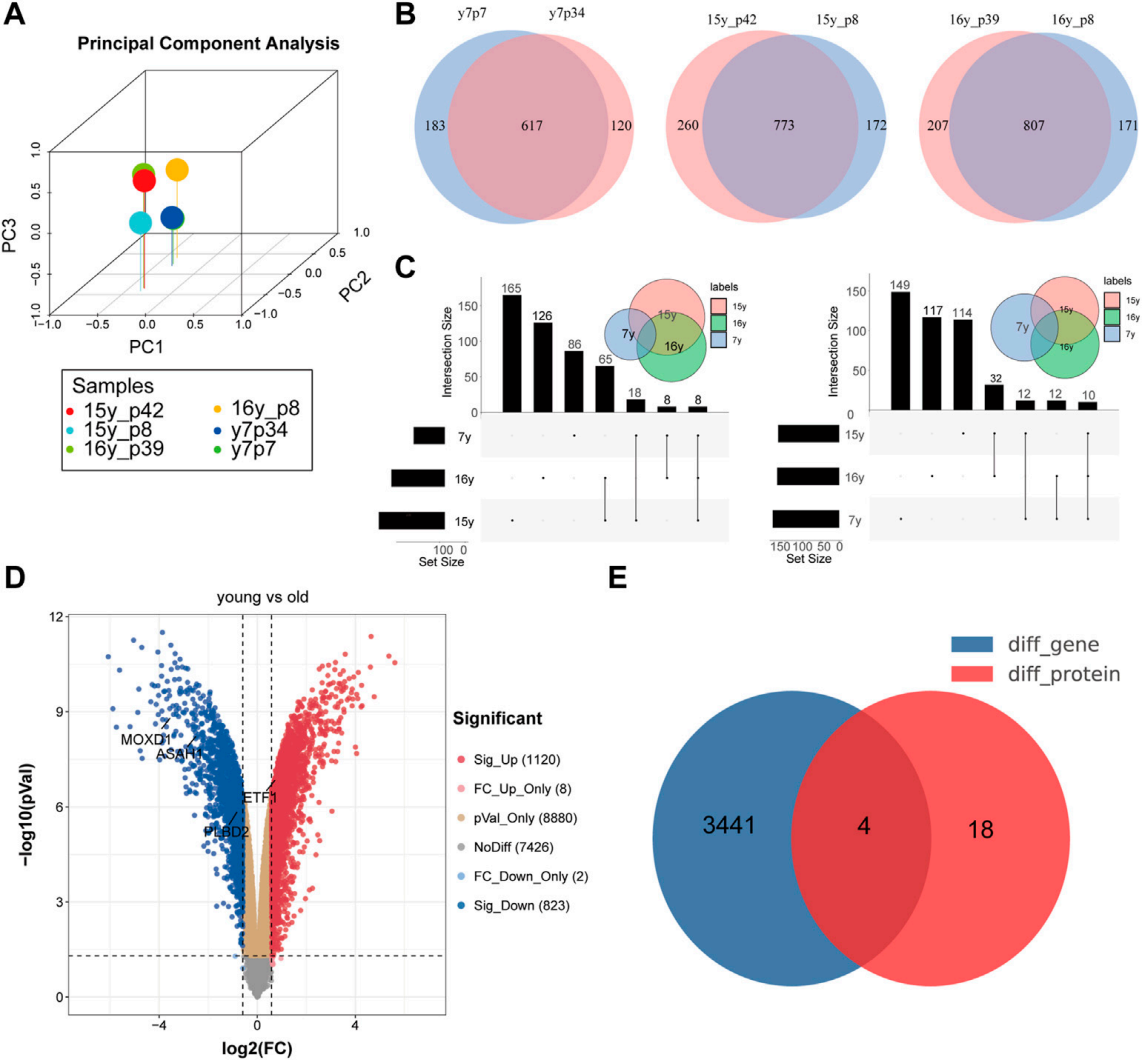
Identification of differentially expressed genes in transcriptome and proteome at the same time. **(A)**. PCA analysis showed that there was no deviation from the 6 samples in the protein group; Identification of co-existing molecules in different data sets by VENN **(B)** and UPSET **(C)** analysis; **(D)**. Volcanic map of difference analysis; **(E)**. Identification of differentially expressed genes in transcriptome and proteome at the same time.

PCA showed that the expression patterns of 6 samples (*proteomics* from 3 senescence and 3 young fibroblasts) were normal and there were no outlier samples (Figure 2A). Because of the qualitative data, the specific expression can not be obtained, so we judge whether a specific molecule is differentially expressed between the two groups according to the expression of a specific molecule in the experimental group (or not) (Figure 2B). Through the comparative analysis of the aging group and the young group in the proteomic matrix, we found that 8 proteins (ASAH1, CAMK2D, CD59, GPX1, MOXD1, PLBD2, PPP1R14B, and VASP) were not detected in the young group, but detected in the senescence group, while 10 proteins (COL1A1, ETF1, MMP14, P4HA2, RHOG, RPL31, SLC38A2, TUBAL3, RSL1D1, and G3BP1) were not expressed in the senescence group, but expressed in the young group (Figure 2C). Proteomic data are presented in Supplementary Table S1. In addition, using the expression matrix of the 16th generation fibroblasts and the 74th generation fibroblasts in the GSE63577 data set, we found that 1,220 genes were highly expressed in the young fibroblasts, while 823 genes were highly expressed in the senescence fibroblasts (Figure 2D). By comparing and combining the differential molecules in proteome and transcriptome, we identified four molecules (ASAH1, CAMK2D, PLBD2, and ETF1) as SRMs in fibroblast (Figure 2E).

### Four key Senescence-Related Molecules Are Significantly Associated With Aging-Related Pathways

**FIGURE 3:**
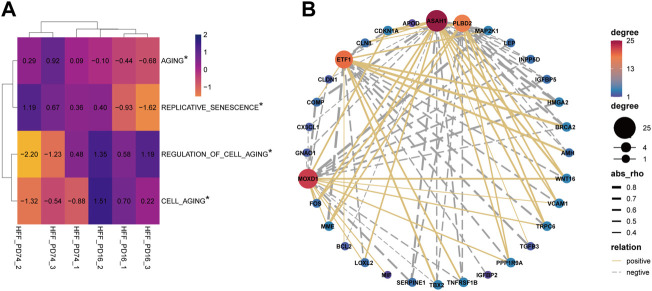
Explore the relationship between four SRMs and aging. **(A)**. GSVA confirmed that there were significant differences in the pathways of senescence and cell senescence between the young group and the old group; **(B)**. Analysis of the relationship between four SRMs and aging genes. The yellow solid line represents positive correlation, the gray dotted line represents negative correlation, and the thicker the line, the stronger the correlation.

The results of GSVA suggest that there are significant differences in aging-related pathways between the young group and the senescence group (Figure 3A). Next, 304 genes in aging-related pathway were detected and 41 of them were significantly differentially expressed between the aging group and the young group (adjust-pvalue<0.05, |FC|>2). Then we analyzed the pearson correlation between SRMs and 41 differential aging genes in the training set GSE63577 (Figure 3B) and the validation set GSE64553 (Supplementary Figure S1B), respectively. We find that PLBD2 has a significant positive correlation with WNT16 and CDKN1A and a significant negative correlation with HMGA2 in both training set and validation set. What’s more, in the two data sets, ASAH1 was significantly negatively correlated with HGMA2 and TNFRSF1B, but positively correlated with WNT16, CDKN1A, and LOLX2. ETF1 was negatively correlated with CDKN1A, but positively correlated with HMGA2. Finally, there is a significant positive correlation between MOXD1 and WNT16 and FOS in the two datasets. Crucially, in the GSE64553 data set, the expression of four SRMs was also significantly different between the aging group and the young group (Supplementary Figure S1A).

### Senescence-Related Molecules Are Closely Related to Immune Cells Infiltration

**FIGURE 4:**
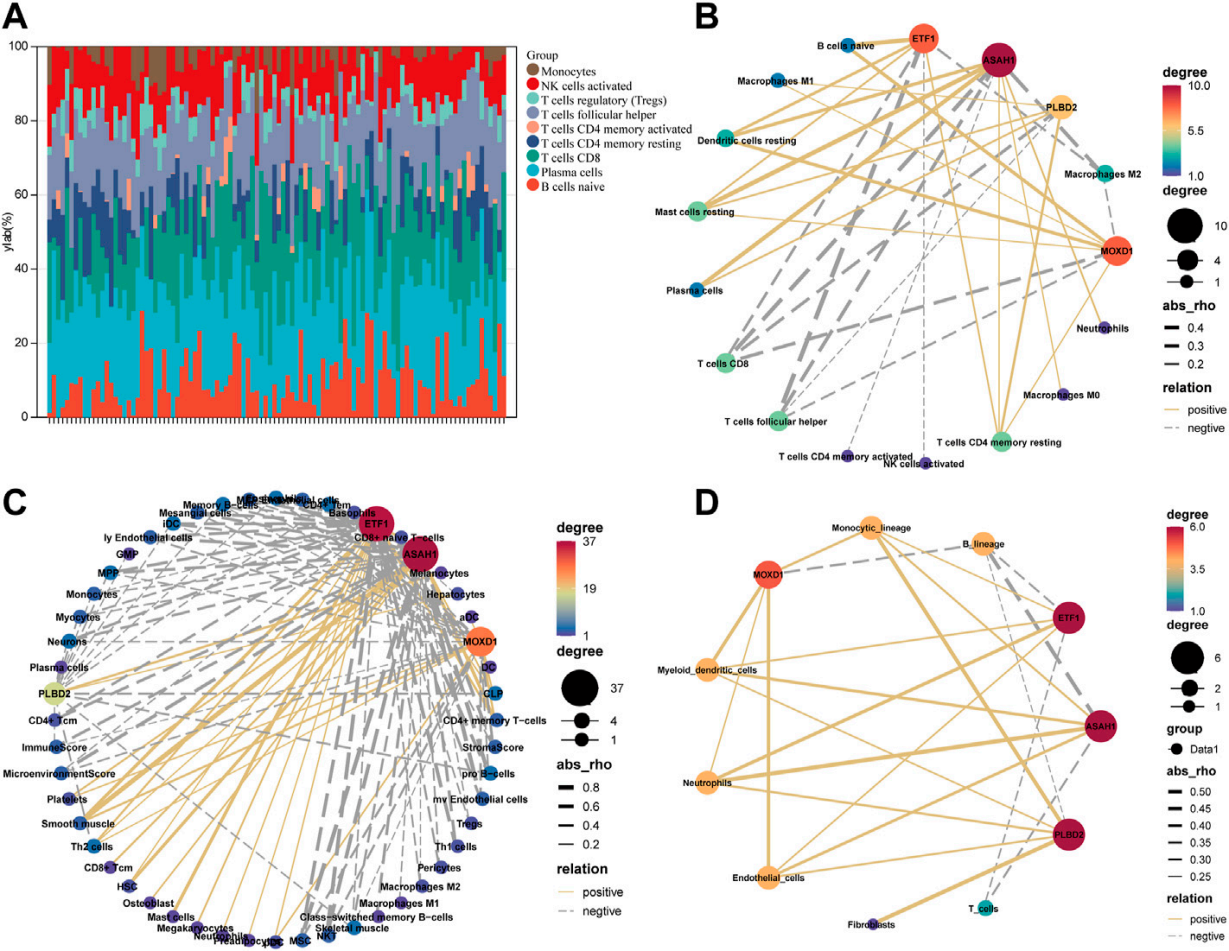
Explore the relationship between four SRMs and immune cell. **(A)**. The landscape of immune cell infiltration in the tissue was calculated by CIBERSORT. **(B)**. The correlation between the expression of four SRMs and the abundance of immune cell infiltration in tissue, which was calculated by CIBERSORT. **(C)**. The correlation between the expression of four SRMs and the abundance of immune cell infiltration in tissue, which was calculated by Xcell. **(D)**. The correlation between the expression of four SRMs and the abundance of immune cell infiltration in tissue, which was calculated by MCPcounter. The yellow solid line represents positive correlation, the gray dotted line represents negative correlation, and the thicker the line, the stronger the correlation.

We eliminate the batch effect of GSE18876 and GSE85358 datasets through the “SVA” package, and merge them into one dataset (Supplementary Figure S2A). There are 11164 common genes between the two datasets (Supplementary Figure S2B). In addition, there were significant differences in the expression of SRMs in the transcriptome of dermis (Supplementary Figure S2C). Next, according to the gene expression in the fusion matrix, we calculated the infiltration abundance of immune cells in the skin tissue by CIBERSORT (Figure 4A), MCPcounter, and Xcell. According to the expression of SRMs and the abundance of immune cell infiltration, we calculated the person correlation between them (Figures 4B–D). The results suggest that ETF1, PLBD2, MOXD1, and ASAH1 have a significant negative correlation with immune cells related to T cells CD8, T cells follicular helper, T cell CD4 memory activated, NK cells activated and Macrophages M2, but has a significant positive correlation with immune cells such as T cells CD4 memory resting, Dendritic cells resting, and Mast cells resting.

### ETF1 May Play a Role in the Aging Process by Regulating Immunity

**FIGURE 5:**
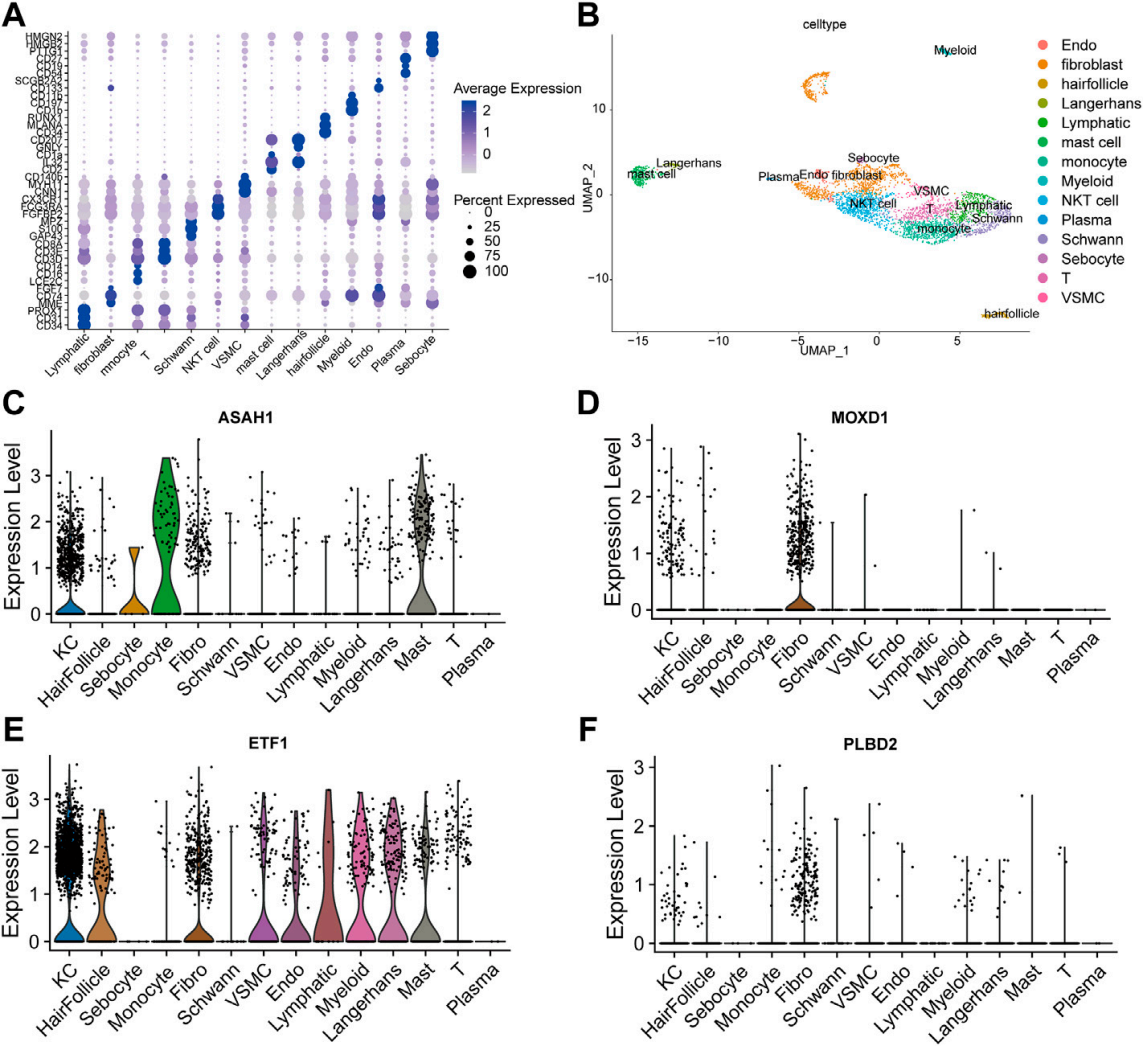
Single cell data analysis. **(A)**. Cell specific biomarkers in skin tissue can well distinguish cell subsets. **(B).** The umap map shows that the integrated data containing five normal skin tissue samples are divided into 14 cell clusters by specific biomarkers of skin tissue cells. Examined the expression of four SRMs in the dermis single cell dataset GSE151177 and found that four SRMs were highly expressed in fibroblasts **(C–F)**.

**FIGURE 6:**
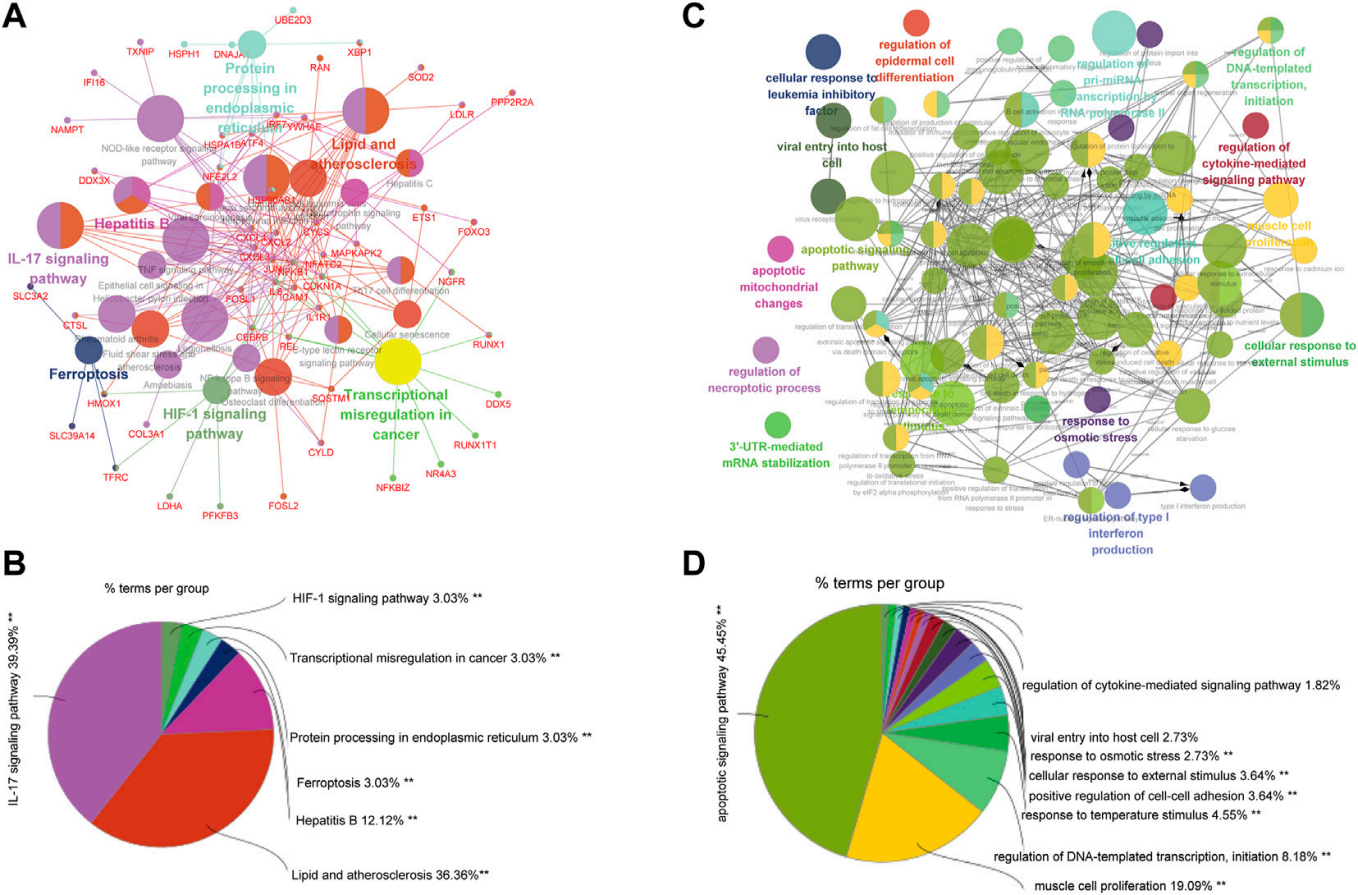
GO and KEGG enrichment analysis of genes significantly related to ETF1. **(A)**. KEGG enrichment analysis of genes significantly related to ETF1; **(B)**. The proportion of each pathway in the results of KEGG enrichment analysis; **(C)**. GO-BP enrichment analysis of genes significantly related to ETF1; **(D)**. The proportion of each pathway in the results of GO-BP enrichment analysis.

**FIGURE 7:**
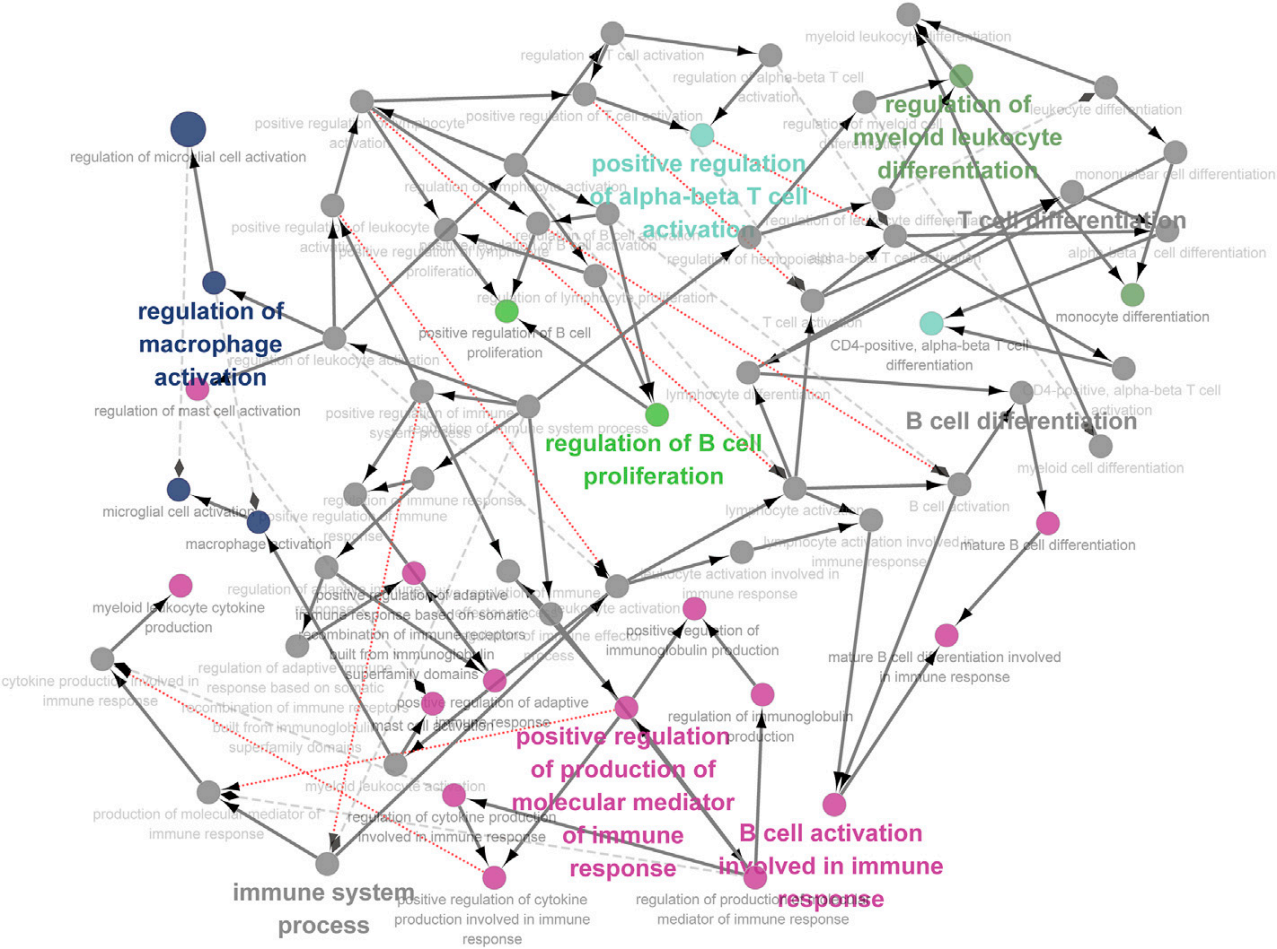
Immune pathway analysis of genes significantly related to ETF1. In order to explore the relationship between ETF1 and immune system, I conducted immune pathway enrichment analysis of genes significantly related to ETF1. The results suggest that a large number of immune-related pathways, including T cell differentiation, B cell differentiation, and immune system processed, are enriched.

We integrated all normal skin samples (a total of 5 cases) in GSE151177 data set for analysis. After three steps of strict initial quality control screening (Supplementary Figure S3), the results showed that we stratified the integrated data into 14 types of cells according to the biomarkers of various cells in skin tissue, including fibroblasts (Figures 5A,B). We examined the expression of SRMs in the dermis single-cell sequencing dataset GSE151177 and found that SRMs were highly expressed in fibroblasts (Figures 5C–F). After annotation, we obtained the expression matrix of 1,162 fibroblasts. Next, we screened the genes significantly related to SRMs (|R|>0.15, *p* < 0.05) and analyzed the functional enrichment of these genes. The results of KEGG and GO-BP enrichment analysis of 151 genes significantly related to ETF suggested that the enrichment pathway was mainly concentrated in IL-17 signaling pathway (Figures 6A,B) and apoptotic signaling pathway (Figures 6C,D). This suggests that ETF1 may play a role in aging through immune-related genes. In order to explore the relationship between ETF1 and immune system, I conducted immune pathway enrichment analysis of genes significantly related to ETF1. The results suggest that a large number of immune-related pathways, including T cell differentiation, B cell differentiation, and immune system processed, are enriched (Figure 7). PPI analysis of 151 genes showed that there may be direct interaction between ETF1 and immune related molecules such as CXCL1, CXCL3, CDKN1A, and IL6, thus regulating immunity (Supplementary Figure S4).

### MOXD1, ASAH1, and PLBD2 Are Closely Related to Skin Aging

36 genes significantly related to MOXD1 were analyzed by GO-BP enrichment analysis. the results showed that regulation of Wnt signaling pathway, collagen fibril organization, and supramolecular fiber organization pathways were enriched (Supplementary Figure S5). In addition, PPI analysis showed that MOXD1 might interact directly with STAR molecules of Wnt pathway and collagen production pathway such as WNT5A, COL3A1, TNN, COL1A1(Supplementary Figure S6). Next, the molecules significantly related to ASAH1 are mainly enriched in epithelium development, tissue development and other pathways (Supplementary Figure S7). Finally, the molecules significantly related to PLBD2 are mainly enriched in HIF-1 signaling pathway and ficolin-1-rich granule lumen pathway (Supplementary Figure S8).

### PCR Validation of the Expression of Senescence-Related Molecules

**FIGURE 8:**
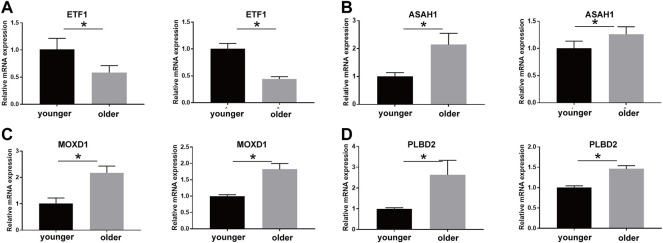
qPCR technique was used to detect the expression of ETF1 **(A)**, ASAH1 **(B)**, MOXD1 **(C)**, and PLBD2 **(D)** in fibroblasts of young and old generations, and the differences were compared. * means the *p* value is less than 0.05.

We validated the expression of SRMs by qRT-PCR experiment using the senescence model of passage fibroblasts. The results showed that the expression of MOXD1, PLBD2, and ASAH1 was up-regulated in the aging group, while the expression of ETF1 was down-regulated in the aging group, which was consistent with the difference between the protein group and the transcriptional group (Figure 8).

## Discussion

In this study, we first combined proteomics and transcriptome to identified four *key SRMs*. Then the correlation between *SRMs* and aging-related pathways was excavated and validated. Next, we validated the expression of *SRMs* at the tissue level and qPCR, and explored the correlation between them and immune infiltrating cells. Finally, at the single-cell transcriptome level, we validated their expression and explored the possible pathway by which they lead to aging.

Eukaryotic translation termination factor 1 (ETF1) encodes a class-1 polypeptide chain release factor. The encoded protein plays an essential role in directing termination of mRNA translation from the termination codons UAA, UAG and UGA. In our study, there was a significant negative correlation between ETF1 and the famous aging promoting gene CDKN1A (p21) (Sharpless and Sherr, 2015). What’s more, it has a significant positive correlation with the famous aging inhibitory gene HMGA2 (Zhu et al., 2013). Surprisingly, ETF1 was significantly down-regulated in both senile passage cells and aging skin tissue. Data from single-cell groups showed that ETF1 was significantly associated with tendentious and inflammatory factors such as CXCL1 (De Filippo et al., 2013), CXCL2, CXCL3, NFKB1(Fock et al., 2010), JUN(Weitzman, 2002), and IL-6 (Eckmann and Neish, 2011). We later analyzed its relationship with inflammation and immunity and found that most of the genes significantly related to it were enriched in IL-17 signaling pathway, Th17 cell differentiation pathway, Cellular senescence, autophagy signaling pathway, and immunomodulatory pathways. IL-17 can significantly reduce the expression of P21, and Th17 cells secreting IL-17 can induce fibroblast senescence (Faust et al., 2020). Therefore, we speculate that ETF1 may affect the changes of inflammatory factors such as IL-17, IL-6, and NFKB1 by indirectly regulating the enrichment and differentiation of immune cells. Autophagy is closely related to aging. The diversity of cell functions of different types of autophagy and the interaction between autophagy and other aging determinants are putting autophagy at the center of the aging process (Kaushik et al., 2021). ETF is significantly associated with 34 autophagy-related molecules, including ATF4 (Rutkowski and Kaufman, 2003), so ETF1 is likely to affect aging through autophagy.

MOX (monooxygenase X), is a member of the copper monooxygenase family that includes dopamine beta-monooxygenase (DBM) and peptidylglycine alpha-hydroxylating monooxygenase (PHM) (Xin et al., 2004). As a member of MOX, Monooxygenase DBH like 1 (MOXD1) has not been studied in the field of aging. In our study, MOXD1 was positively correlated with senescence and cell cycle famous molecule WNT16 (Binet et al., 2009) and FOS(Jiang et al., 2021), and the molecules COL1A1, KREMEN1, PTK7, SEMA5A (D'Aguanno et al., 2018), TNN, and WNT5A (Kawarazaki et al., 2020), which were significantly positively related to MOXD1 were mainly enriched in WNT signal pathway and collagen fiber formation pathway. PPI analysis showed that MOXD1 might interact directly with WNT5A, COL3A1, TNN, and COL1A1. In addition, compared with non-aging fibroblasts and non-aging skin tissues, the expression of MOXD1 in aging fibroblasts and tissues were significantly higher. Therefore, we speculate that the high probability of MOXD1 may regulate senescence by affecting the WNT pathway and changing the cell cycle.

N-acylsphingosine amidohydrolase 1 (ASAH1) encodes a member of the acid ceramidase family of proteins. It is essential for the formation of mature lysosomal enzymes, which are overexpressed in many human cancers and may play a role in cancer progression (Robak et al., 2017). Rachel et al. found that ASAH1 increased highly in senescent cells. Silencing ASAH1 in pre-senescent fibroblasts decreased the levels of senescence proteins p16, p21 and p53, and decreased the activity of β-galactosidase related to senescence. ASAH1 promotes senescence, protects senescent cells, and endows it with resistance to anti-aging drugs (Munk et al., 2021). In this study, the expression of ASAH1 was up-regulated in aging fibroblasts and aging skin tissues. Correlation analysis shows that ASAH1 was significantly negatively correlated with HGMA2 and TNFRSF1B, but positively correlated with Well-known cell cycle related genes WNT16, CDKN1A, and LOXL2 (Kim et al., 2014). GSTK1, DCN, CTSB, and SFRP2, which are significantly positively related to ASAH1, are enriched in tissue development pathway and epithelial development pathway, suggesting that ASAH1 may affect development and regulate the phenotype of aging by affecting cell cycle related genes.

Little research has been done on phospholipase B domain containing 2 (PLBD2, also known as P76) in human tissues. In the present research, PLBD2 was significantly up-regulated in aging tissues and fibroblasts and was significantly positively correlated with p21 and WNT16. In addition, the molecules significantly related to PLBD2 are mainly enriched in HIF-1 signaling pathway and ficolin-1-rich granule lumen pathway. Although there is no previous research guidance, our work suggests that PLBD2 may play a key role in the aging process.

## Conclusion

In general, based on the analysis of proteomics and transcriptome, we identified SRMs that may affect aging and predicted their possible mechanisms, which provides a new target for preventing aging, especially skin aging.

## Data Availability

The datasets presented in this study can be found in online repositories. The name of the repository and accession numbers can be found below: iProX; IPX0004404000.

## Abbreviations

GEO, gene expression omnibus; SRMs, senescence-related molecules; PCA, principal component analysis; ETF1, eukaryotic translation termination factor 1; ASAH1, N-acylsphingosine amidohydrolase 1; MOXD1, monooxygenase DBH like 1; PLBD2, phospholipase B domain containing 2.
