# Supplementary material for: Identification of Four Biomarkers of Human Skin Aging by Comprehensive Single Cell Transcriptome, Transcriptome, and Proteomics
Source: Front Genet. 2022 Aug 23;13:881051. doi: 10.3389/fgene.2022.881051 (PMC9445490; doi:10.3389/fgene.2022.881051)
Supplement: Supplementary file 1 [file Table1.DOCX]

**Supplementary Figures**

**
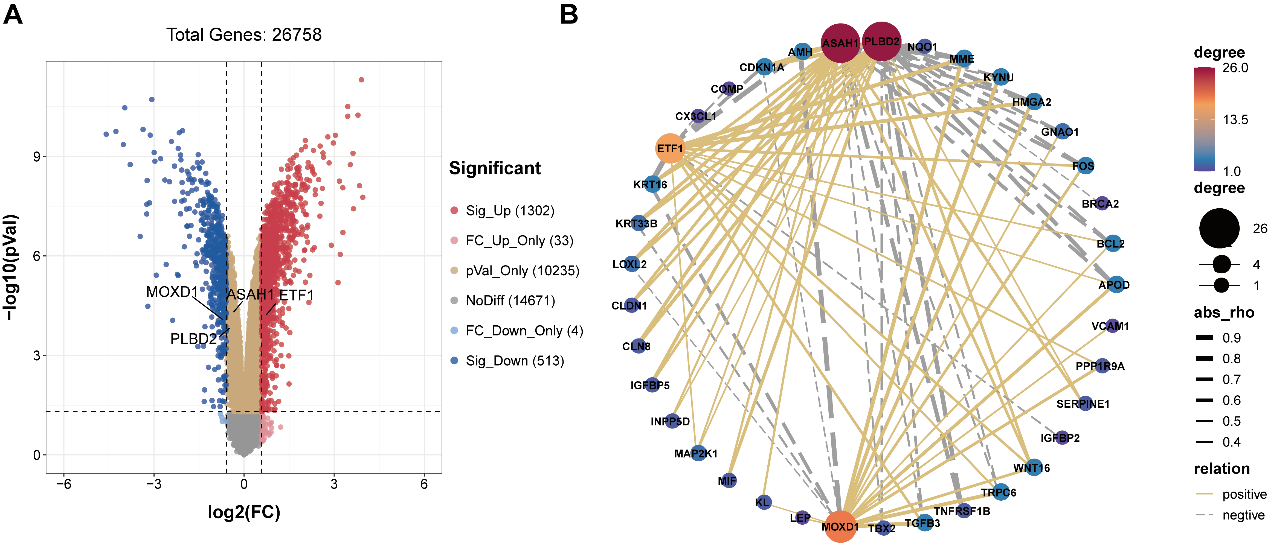
**

**Figure S1.** Validation of the differential expression of four molecules and their correlation with aging genes in GSE64553 dataset. **A**. Volcanic map of difference analysis; **B**. Analysis of the relationship between four genes and aging genes.

**
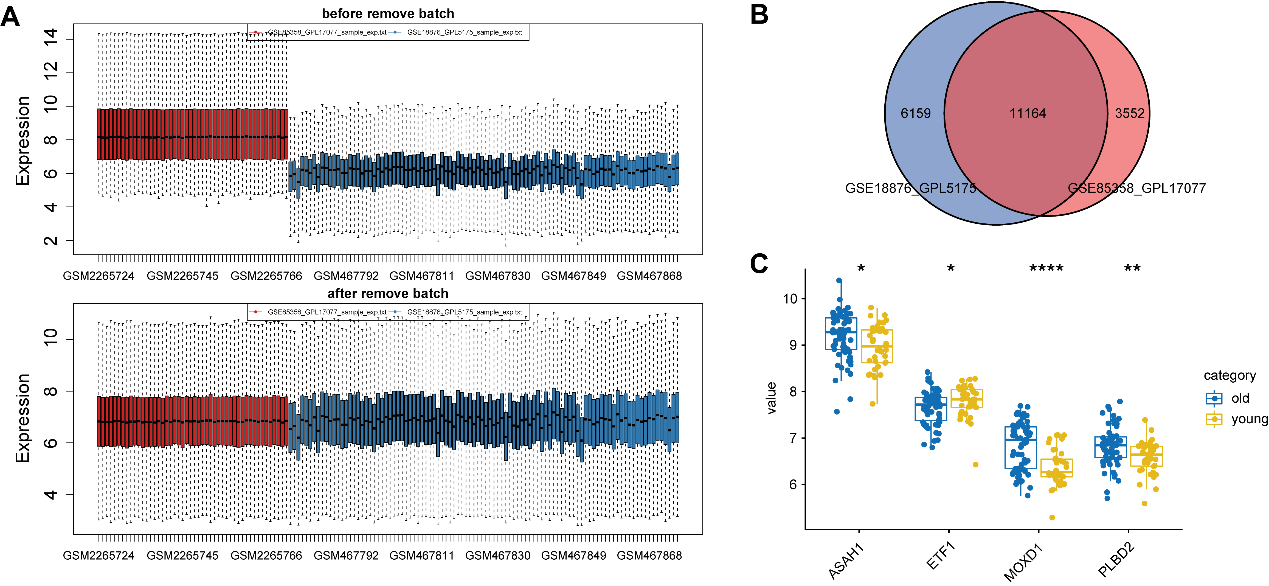
**

**Figure S2.** Validation of differential expression of four molecules in dermis. **A**. eliminate the batch effect of GSE18876 and GSE85358 datasets through the "SVA" package. **B**. there are 11164 common genes between the two datasets. **C**. there were significant differences in the expression of four key molecules in the transcriptome of dermis.


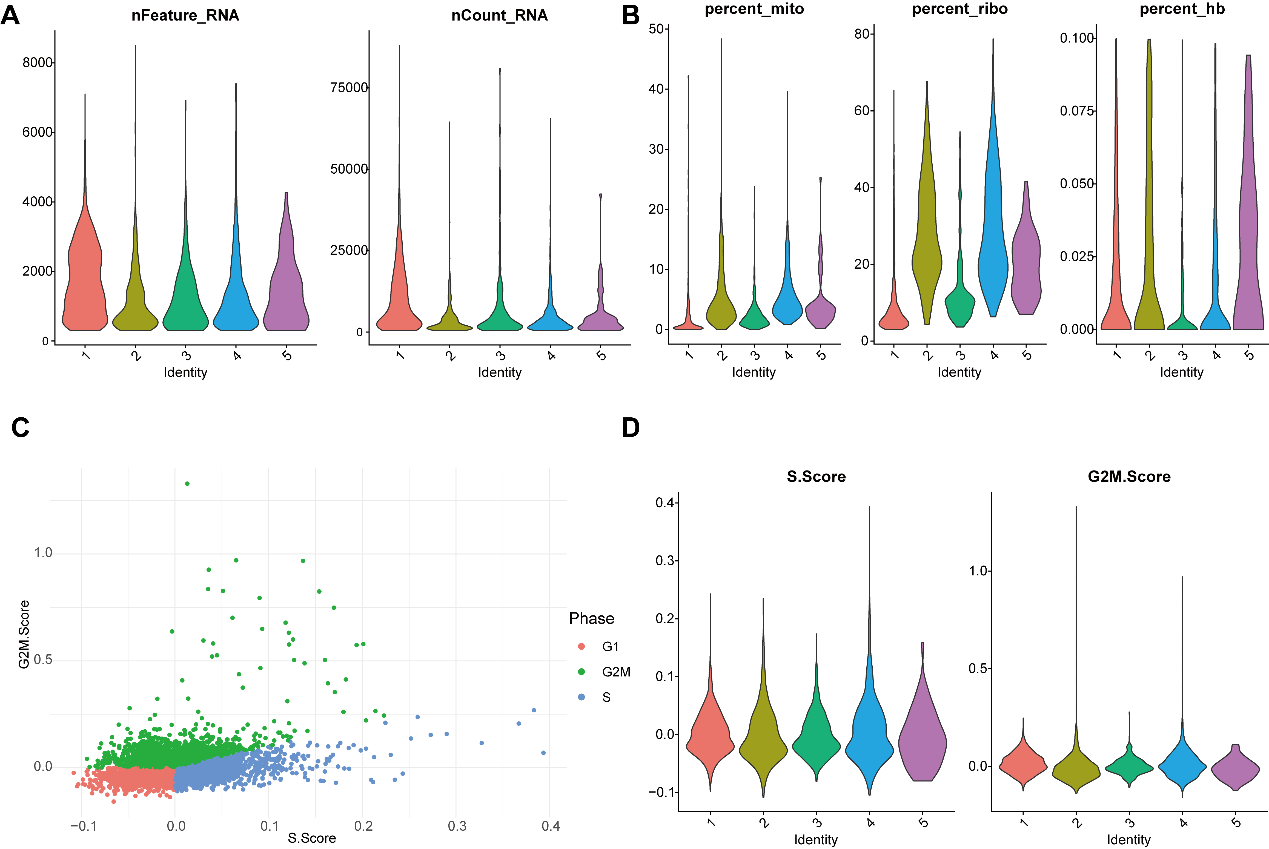


**Figure S3.** In the quality control stage, we filtered out the cells with the minimum number of expressed genes less than 300 and the genes with the minimum number of expressed cells less than 4 (**A**). Then we conducted the second screening according to the conditions that the proportion of mitochondrial genes is less than 50% and the proportion of ribosomal genes is more than 3% (**B**). Finally, we filtered out the housekeeping gene and scored the cell cycle (**C and D**). The number of abscissae in the figure represents 5 samples.


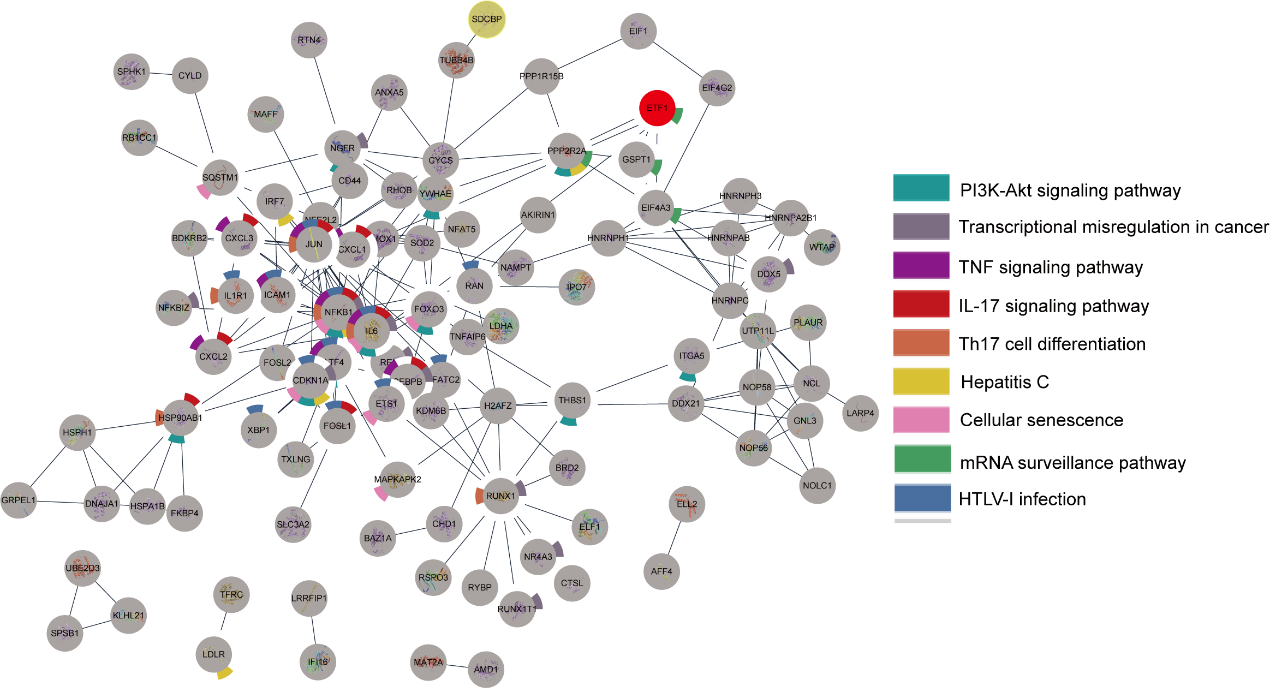


**Figure S4**. PPI analysis of 151 genes showed that there may be direct interaction between ETF1 and immune related molecules such as CXCL1, CXCL3, CDKN1A, and IL6.


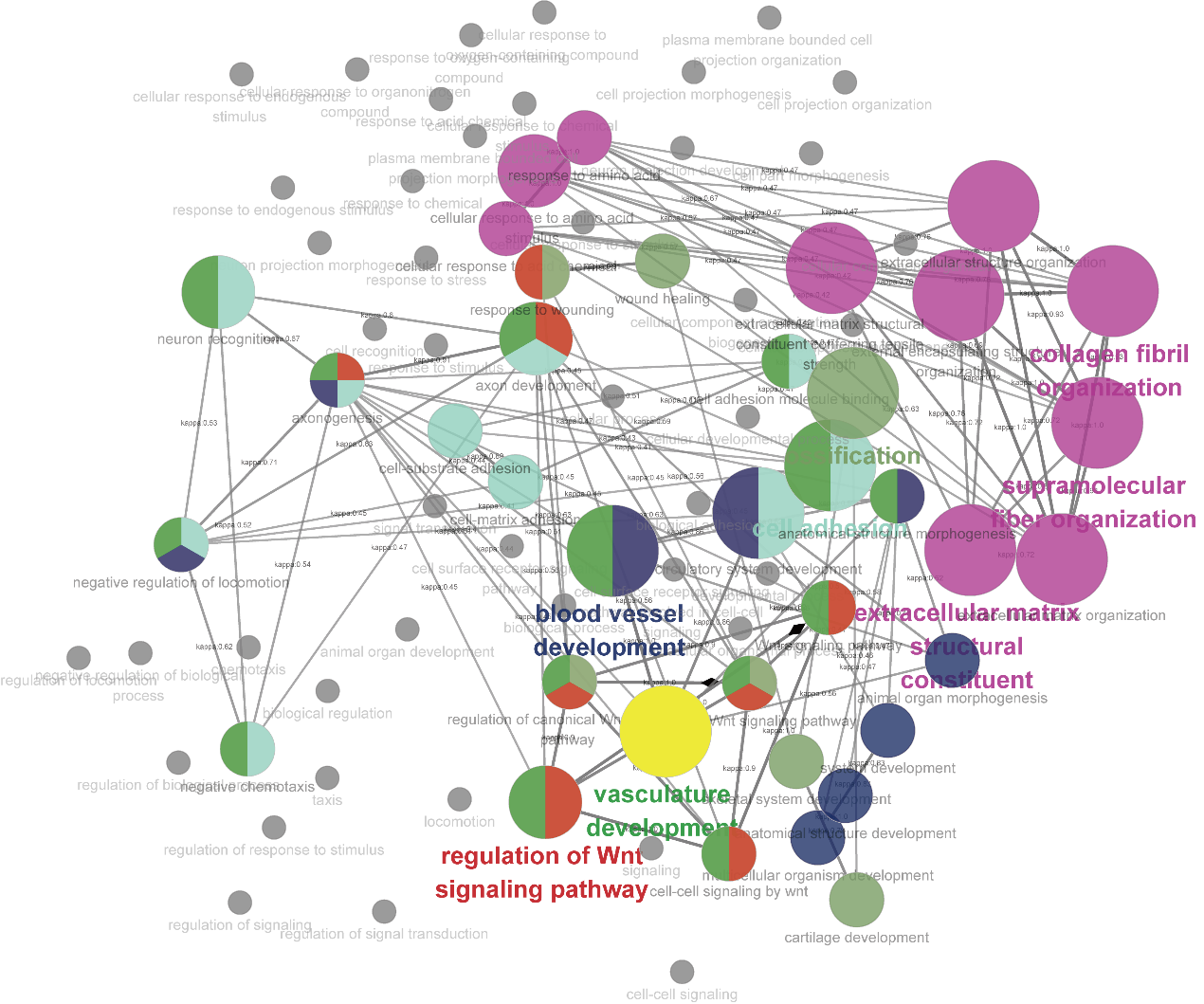


**Figure S5**. 36 genes significantly related to MOXD1 were analyzed by GO-BP enrichment analysis. the results showed that regulation of Wnt signaling pathway, collagen fibril organization, and supramolecular fiber organization pathways were enriched.


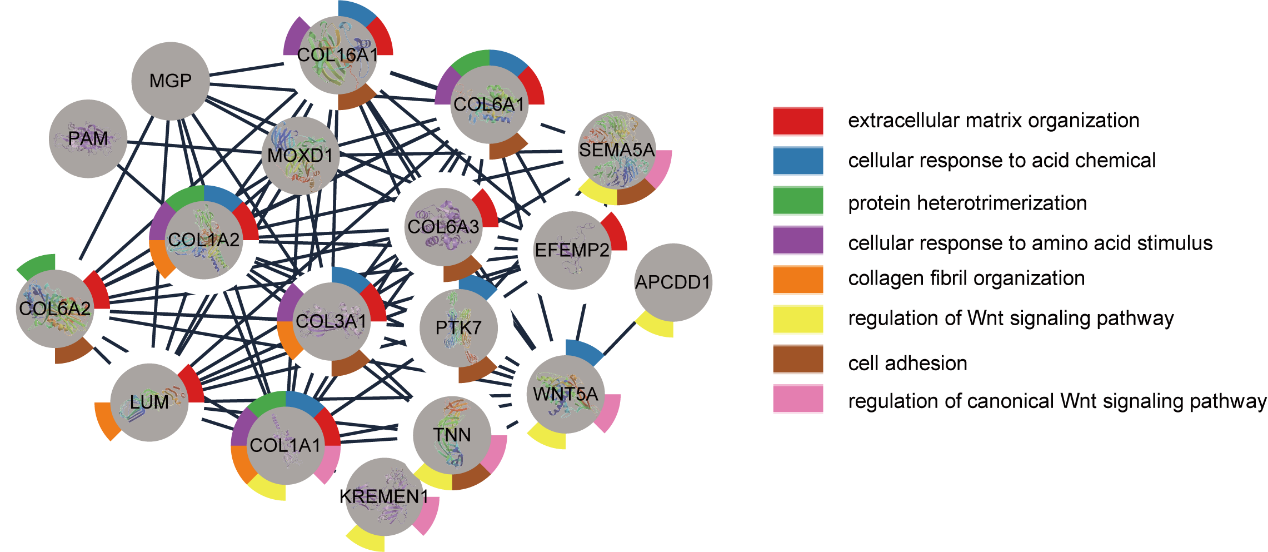


**Figure S6**. PPI analysis showed that MOXD1 might interact directly with STAR molecules of Wnt pathway and collagen production pathway such as WNT5A, COL3A1, TNN, COL1A1.


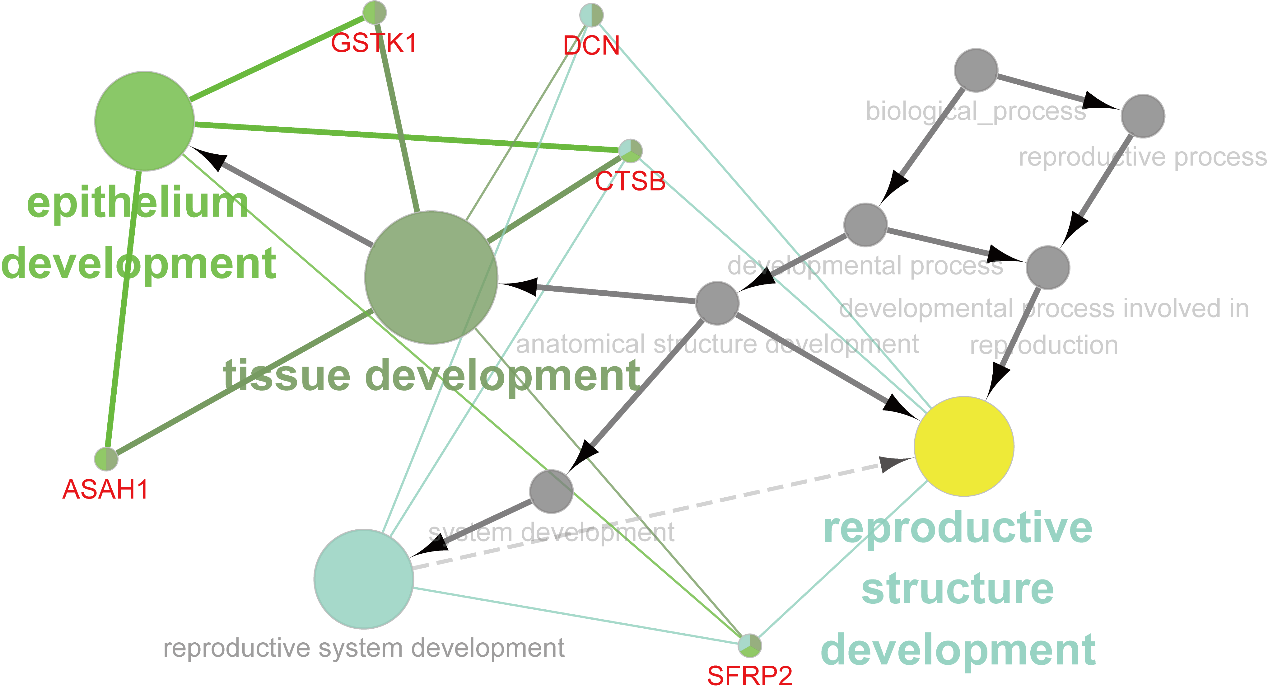


**Figure S7**. the molecules significantly related to ASAH1 are mainly enriched in epithelium development, tissue development and other pathways.


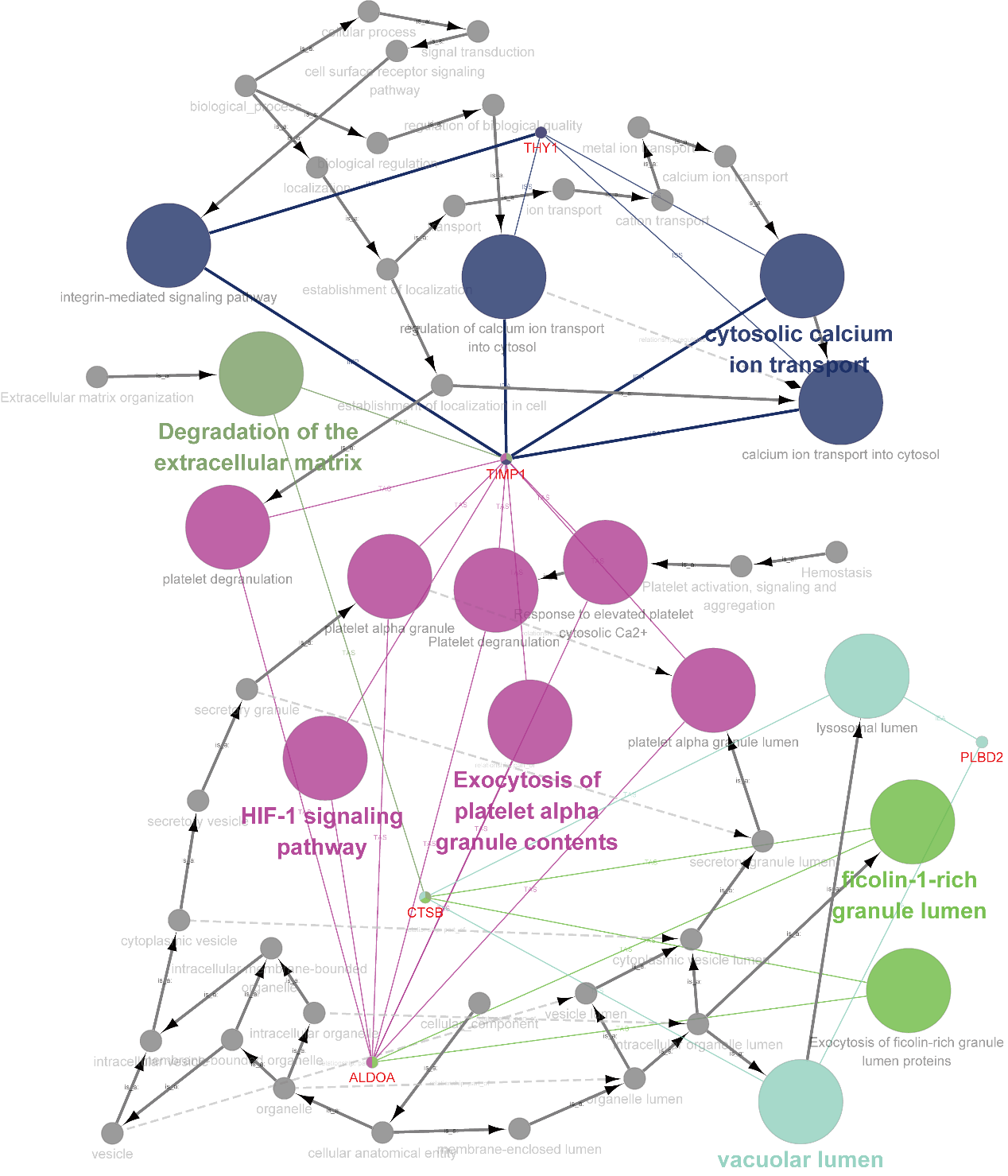


**Figure S8**. the molecules significantly related to PLBD2 are mainly enriched in HIF-1 signaling pathway and ficolin-1-rich granule lumen pathway.
